# Maggot metabolites and their combinatory effects with antibiotic on *Staphylococcus aureus*

**DOI:** 10.1186/1476-0711-10-6

**Published:** 2011-02-07

**Authors:** Shuchi Arora, Carl Baptista, Chu Sing Lim

**Affiliations:** 1 School of Chemical and Biomedical Engineering, Nanyang Technological University, 62 Nanyang Drive, 637459, Singapore; 2 Medifly Laboratories, Biomedical and Pharmaceutical Engineering Cluster, Nanyang Technological University, 637539, Singapore; 3 School of Mechanical and Aerospace Engineering, Nanyang Technological University, 50 Nanyang Avenue, 639798, Singapore

## Abstract

**Background:**

Maggot therapy has been in practice for effective debridement, disinfection and healing of chronic wounds. Due to their antiseptic action during wound healing, their metabolites have been investigated in the past for antibacterial activity. They have been particularly useful for treatment of wounds infected with multi-drug resistant *Staphylococcus aureus*. Antibiotics, on the other hand, can predispose bacteria to develop resistance. Substances that are able to modulate or delay the occurrence of resistance in bacteria are under investigation by many researchers around the world. In the present study, antibacterial activity in excretions/secretions (ES) from maggots of *Lucilia cuprina *blowfly was demonstrated. The extracts were also screened in combination with antibiotic, ciprofloxacin.

**Methods:**

*L. cuprina *blowfly maggots were reared for extraction of its metabolites. The ES extracted was screened against *S. aureus*, alone and in combination with ciprofloxacin, both for short term and long term exposure analysis. A microchannel-based device and system was used for experiments instead of conventional techniques.

**Results:**

The original ES had shown partial bacterial growth inhibition. However, in combination with ciprofloxacin, at sub-inhibitory concentrations, certain combinations revealed anti-staphylococcal activity, with bacterial reduction of up to 50%, after 24 hours. The six day study on *S. aureus *exposed to ES-ciprofloxacin combination suggested a potential delay in development of adaptive resistance as opposed to when ciprofloxacin was used as single agent.

**Conclusions:**

The combination effect of ES and ciprofloxacin at sub-MIC levels showed enhanced antibacterial activity compared to the effect of ES and ciprofloxacin as single agents. Based on the results of ES-ciprofloxacin combinations, a more effective means of treatment for *S. aureus *can be proposed.

## Background

Maggot therapy has been traditionally practiced for debridement of necrotic wounds as well as for curing bacterial infections at the wounds site [1,2]. It has been reported to have advantages over the conventional methods, especially for treatment of wounds infected by multi-drug resistant methecillin-resistant *Staphylococcus aureus *(*S. aureus*) or MRSA [3-8]. Their antiseptic action has been investigated by many researchers in the past for specialized antibacterial properties or presence of antimicrobial factor(s). In particular, it has been under investigation since 1930s. Simmons *et. al. *[9,10], first studied the mechanism of action of maggot disinfection on wounds. They found that the excretion of maggots exhibited a strong and rapid disinfection action on *S. aureus*. Subsequently, several other groups collected the excretions/secretions (ES) of maggots and screened it on various microbes [11-15]. Many species of blowfly maggots were investigated against both gram-negative and gram-positive bacteria [11,12,16]. However, most of these investigations were carried out using the maggot of *Lucilia sericata*, a species of blowfly used for maggot therapy in Europe and Americas. For the present study, investigations were performed on ES extracted from maggots of *Lucilia cuprina*, acquired in Singapore.

The disinfection action of the maggots was suggested by some to be present in the excretions from the maggots [9,14], while others reported ingestion and gut activity [17]. Recently, a low molecular weight insect peptide was found mainly responsible for the antimicrobial activity of the maggots, when exposed to an infectious environment of a wound. The peptide, lucifencin, that belongs to the insect antimicrobial peptides (AMP), defensin family, was reported to be secreted in the hemolymph, body fat and ES of the maggots. The maggots were raised in an environment simulating a wound that increased the production of the AMP as an innate immune response of the insect [18,19].

The antibacterial activity of *L. cuprina *maggot ES was previously shown to have partial growth inhibition of gram-positive, *S. aureus *[20]. In the present study, the maggot ES was analysed for presence of an antibacterial factor using gas chromatography-mass spectrometry (GC-MS) and pH test. Furthermore, with an aim to enhance antibacterial activity, ES extractions were combined with ciprofloxacin at sub-minimum inhibitory concentration (MIC) levels and screened against *S. aureus*. Ciprofloxacin is a broad spectrum fluroquinolone antibiotic, commonly used for treatment of bacterial infections. There have been a number of instances of increasing ciprofloxacin resistance in *S. aureus *reported in the past [21,22]. The combination investigations of the antibiotic with natural extracts of the insect was performed over an extended period of time, to study their effects on the rapid adaptive resistance in *S. aureus *for ciprofloxacin. The experiments were carried out on a microchannel-device and a tailored monitoring system [23].

## Materials and methods

### Experimental Set-up

A microfluidic device designed, fabricated and tested for the purpose of drug-mixing and cell culturing was used for the study [23]. The mixing of fluids was based on specialized microchannel geometry. Passive mixing occurred at the wells where the cells were cultured. A system to provide favourable environmental conditions and to monitor cell behaviour was also used along with the device. The incubator incorporated in the system was able to maintain a temperature of 37 ± 0.2°C for extended periods of time. The design principles and testing of the experimental setup has been described previously [23].

### Bacterial strain and media

Gram-positive *Staphylococcus aureus *American Type Culture Collection (ATCC) 29213 (methecillin-sensitive *S. aureus *or MSSA), acquired from ATCC was used for the experiments. The strain was stored in Luria broth containing glycerol (40% v/v) at -80°C. Experimental inocula and stocks were prepared in Iso-sensitist (IS) broth and agar, respectively, acquired from Oxoid (Singapore). The stocks were subsequently stored at 4°C for up to six weeks. For preparation of the maggot ES, phosphate buffer saline (PBS) obtained from Biomedia (Singapore) was used as the medium.

### Antibiotics and MIC determination

Ciprofloxacin (99% purity) obtained from Sigma-Aldrich (Singapore) was used for the experiments. Stock solution with concentration 10 mg/mL of the antibiotic was prepared in sterile deionized water (DI) with 0.1 M NaOH to aid dilution. Aliquots of the stock were prepared and stored at -20°C for further use. On the day of experiment, working solution of the antibiotic was prepared by diluting the stock solution with sterile PBS as media.

The MIC of the antibiotic was determined by the broth microdilution method, explained elsewhere [24,25]. Briefly, concentrations with a starting range of 128 μg/mL to 0.0625 μg/mL of the antibiotic was prepared in wells of a 96-well plate in triplicates (Greiner Bio one, Singapore), and bacterial inoculum were added to this 2-fold dilution series. The plate was incubated at 37°C aerobically for 24 hours. The MIC was then determined as the minimum concentration of the antibiotic in serially diluted assay with no visible bacterial growth. This was re-confirmed in a spectrometer at 600 nm wavelength incident light.

### Maggot ES extraction, analysis and MIC determination

The various techniques used to extract the ES or useful antibacterial extracts from the maggots of *L. cuprina *were previously compared and reported [20]. Based on the bacterial inhibitory results at the end of 24 hours from the start of inoculation of bacteria with maggot extracted ES, the optimum technique was adopted. This technique was implemented in the present study and briefly described in the following paragraph.

**Figure 1:**
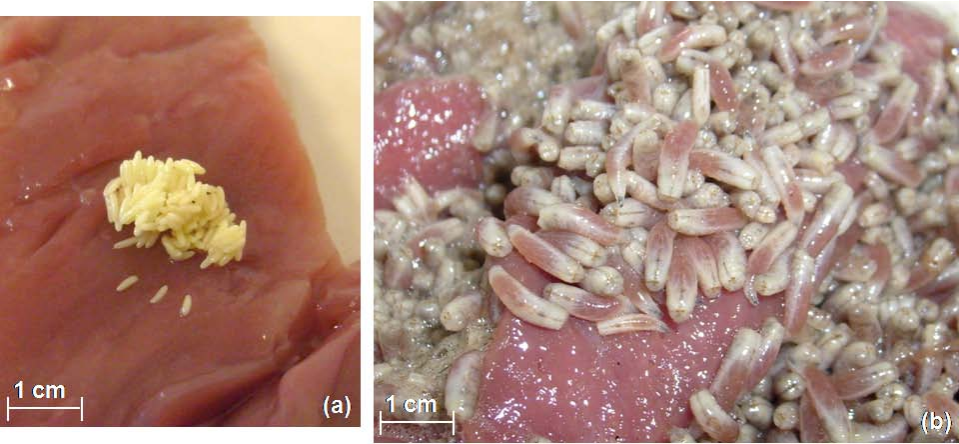
**Rearing of *Lucilia cuprina *maggots**. (a) Maggots eggs. (b) Late second instar maggots obtained at the end of day two of incubation (at 35°C) of eggs on the meat.

*L. cuprina *blowflies were reared on raw meat in plexi-glass cages under controlled humidity and temperature conditions (25°C), at Medifly Laboratories, Nanyang Technological University, Singapore. Eggs laid on the meat were treated with 70% ethanol and sterile distilled water successively three times. The treated eggs were deposited on fresh meat and allowed to hatch to maggots for 2-3 days in an incubator at 35°C (see Figure 1). Late second/early third instar maggots were aseptically transferred to a flat petri-plate and washed with ethanol and sterile distilled water successively three times and soaked in filter paper. Treated maggots were transferred to a 15 mL sterile tube with PBS, to a density of 100 larvae per 200 μL of PBS. They were allowed to incubate in the test tube in dark at room temperature (25°C) for 1 hour. Resultant maggot ES obtained was transferred to another tube using a pipette and autoclaved for 20 minutes at 121°C. Subsequently, the ES was allowed to cool to room temperature. Any remaining ES was stored at -20°C for analysis and future use.

The extracted ES was made to undergo gas-chromatography and mass-spectrometry (GC-MS) analysis to test the presence of previously suggested antibacterial compound, phenylacetaldehyde [26]. The tests were performed on a gas chromatograph (GC, Agilent 7890A) and mass spectrometer (MS, 5975C MSD) using a HP-5MS analytical column. The GC was fitted with a manual splitless injector, which was maintained at a temperature of 250°C. The complete parameters of the GC are summarized in Table 1. The mass spectrometry scanning was done across the range of m/z 35-450. The temperature at the inlet was maintained at 280°C to prevent condensation of the analytes. The parameters for the MSD are summarized in Table 2. The compounds detected by the mass spectrometer were compared in structure to those in the Nist98 database for mass spectral peaks and standard solution peaks.

**Table 1:** Gas Chromatograph control parameters

Gas Chromatograph	Agilent 7890A		
**Analytical Column**	HP-5MS (30 m × 0.25 mm × 0.25 mm)		

**Injection Port Type**	Manual Split/Splitless		

**Injector Temperature**	250°C		

**Pressure**	11.567 psi		

**Injector Type**	Split		

**Split Ratio**	200:1		

**Split Flow**	200 mL/min		

**Gas saver**	OFF		

**Total flow**	204 mL/min		

**Carrier Gas**	Helium		

**Oven program**	**Temperature**	**Hold Time**	**Rate**
	
	40°C	1 min	
	
	280°C	5 min	10°C/min

**Table 2:** Mass spectrometer control parameters

Mass Spectrometer	5975C MSD
**GC inlet line temperature**	280°C

**MS quad temperature**	150°C

**Full scan range**	m/z 35-450

**Solvent delay**	0.1 min

The pH of the collected ES was also measured using a bench top pH meter (Fisher Scientific, Singapore) against PBS reference and standards of pH 7 and 10.

The MIC for the maggot ES was determined similarly, by broth microdilution method using the bacteria and ES mixture by further PBS serial dilution [24].

### Micro-device tests with ES and ciprofloxacin on *S. aureus*

**Figure 2:**
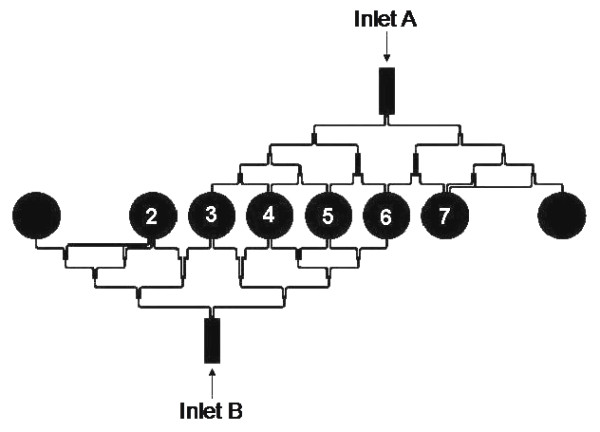
**Microfluidic device: Micro-device with well and inlet markings**.

Overnight MSSA cultures were sub-cultured in IS broth to a standard inoculum of 0.5 Macfarland turbidity [25]. 50 μL of this inoculum was added to 200 μL of sterile ES (collection explained in the previous section) and vortexed thoroughly. The mixture was injected into a sterile micro-device from both the inlets to fill the six wells (wells 2 to 7, see Figure 2) to 25 μL. Subsequently, ciprofloxacin at a working concentration equal to the MIC, determined earlier, was injected from one inlet and sterile PBS from the other, simultaneously, filling each well to a total volume of 50 μL. The resultant antibiotic concentration in the wells of the microchip was then 100%, 80%, 60%, 40%, 20% and 0% of ciprofloxacin MIC (see Figure 2). The inoculated micro-device was incubated in the box incubator of the system at 37°C for 24 hours [23]. Optical density (OD) was measured at the start of the experiment, then after 8 hours and finally at 24 hours. The OD was later translated to bacterial count in CFU/mL using the translation OD-cell count curve previously determined for MSSA in similar experiments [20]. The starting bacterial concentration was determined by performing plate counts of viable colonies at the start of the experiment and then after 24 hours. The cell count (in CFU/mL) was plotted against time to seek a combination of the antibiotic and ES that showed enhanced antibacterial activity compared to ciprofloxacin alone at the same concentration without ES.

A control was also performed without ES simultaneously on a separate device. The antibiotic and PBS were similarly injected from the inlets of device, so that each well had similar ciprofloxacin concentrations explained earlier, but without the ES. OD and plate counts were recorded at the same time intervals as in previous experiments.

Plate counts obtained at 24 hours were compared with the initial bacterial concentration and a relative reduction of viable bacteria after incubation with drugs was determined. The results were plotted against concentration of ciprofloxacin, with and without ES. The results were also compared to each other and with the control in decreasing concentration of ciprofloxacin.

To assess the effects of ES on development of adaptive ciprofloxacin resistance in *S. aureus*, the bacteria was exposed to ciprofloxacin at MIC concentration, with and without the addition of ES, continuously for six days (144 hours). The experiments were performed in the micro-device, by injection of ES in PBS followed by ciprofloxacin (= MIC) from both inlets. The resultant solution in each well of the micro-device would thence have same concentration of ES and ciprofloxacin. The control was prepared separately in 96-well plate for comparison of results. The exposed inocula were analysed after every 24 hours, by diluting and plating 10 μL of sample from each well and counts were averaged. The drugs and media were replenished after every 24 hours.

## Results

### MIC of ciprofloxacin and Maggot ES

The bacteria showed susceptibility to ciprofloxacin at concentration 1 μg/mL. However, there was no visible antibacterial activity observed in the diluted ES forms in the MIC assay, as all the wells were turbid after overnight incubation. However, the original ES was able to show bacterial reduction up to 30% (±10%) from the starting bacterial concentration as also revealed in the time-kill analysis previously conducted [20].

### GC-MS and pH of maggot ES

The qualitative GC-MS analysis of the maggot ES, established the presence of the antibacterial compound, phenylacetaldehyde. The test peaks were compared with those of commercially procured phenylacetaldehyde (≥ 90%, Sigma, Singapore) standard peaks. The quantitative analysis determined an average of 84.5 mL/L (±7.23 mL/L) of the antibacterial compound in the ES. The pH of the ES was in the range of 8.6-8.7 for all samples collected.

### Micro-device drug combinations

The data on bacterial growth in the micro-device was recorded at 0, 8 and 24 hours after MSSA suspension was exposed to drug and drug combinations.

**Figure 3:**
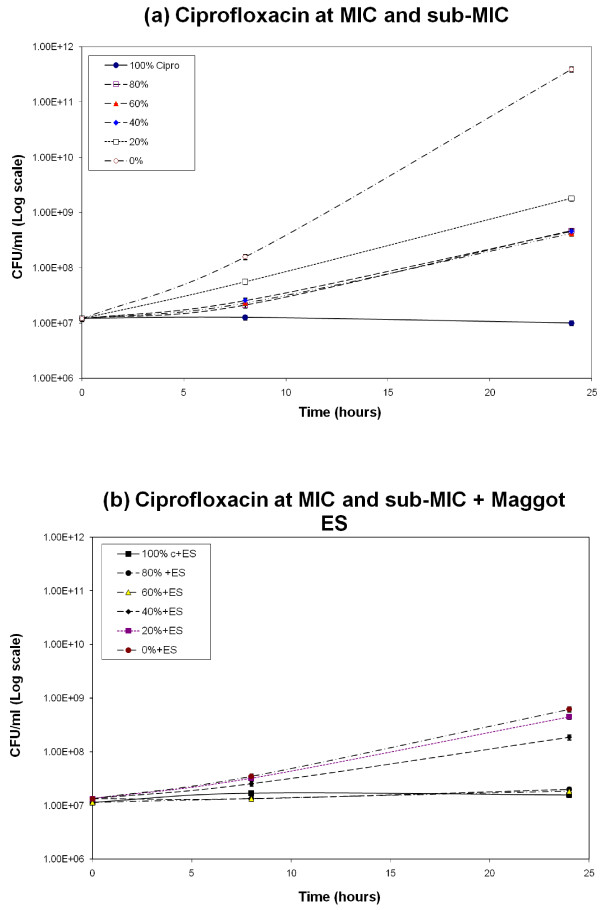
**CFU/mL vs. time functions for *S. aureus *with ciprofloxacin alone and in combination with ES**. Readings directly taken from the micro-device wells. (a) CFU/mL vs. time, when only ciprofloxacin and PBS were injected from the two inlets of the micro-device. (b) CFU/mL vs. time, when ES with bacterial suspension was injected from both inlets, followed by ciprofloxacin and PBS from the inlets of the micro-device. (The coefficient of variability (R^2^) in the predicted data points from the translation of OD to CFU/mL graph was determined by linear regression modeling. The value of R^2 ^was calculated as 0.9024, for all predicted CFU/mL values from recorded OD values). Each calculated data point has ±10% linear error value for cell number in CFU/mL. The OD was recorded in triplicates in three separate micro-devices for each day. The experiments were repeated three times.

The graphs in Figure 3 illustrate CFU/mL versus time functions in different wells of the inoculated micro-device. From the graphs, it can be noted that, with ciprofloxacin at MIC and sub-MIC concentrations, there is significant bacterial growth at all concentrations after 24 hours. The only exception was at 100% concentration, where bactericidal effect was observed. Whereas, with ciprofloxacin in combination with maggot ES, though no significant growth inhibition was observed in Well 7, (100% ES concentration), stunted growth was observed in ES with 80 and 60% ciprofloxacin. Comparing with 60% and 80% ciprofloxacin only, it can be deduced that the addition of maggot ES helped to enhance or potentiate the activity of ciprofloxacin at sub-MIC levels.

**Figure 4:**
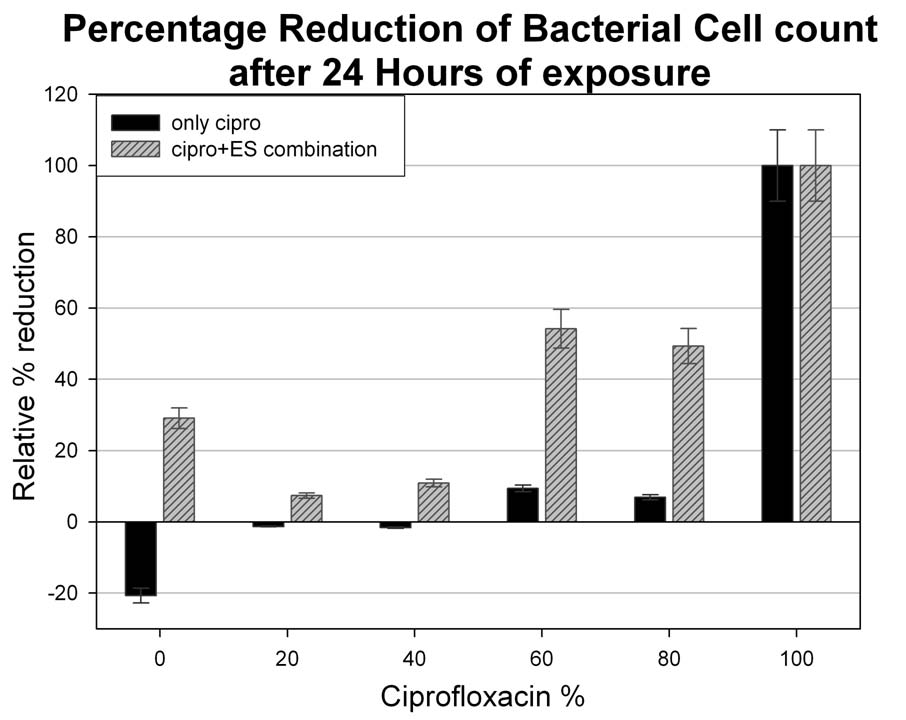
**Percentage reduction graph for 24 hr study**. Percentage reduction from the initial bacterial concentration at '0 hour' calculated after 24 hours of exposure of MSSA to ciprofloxacin at MIC and sub-MIC concentration and maggot ES in combination with ciprofloxacin at various concentrations. The cell number was calculated over a range of dilutions and averaged. Each dilution was plated in triplicates for zero and 24 hours. The combination experiments were repeated seven times with ES collected on the day of experiment.

The micro-device OD results were verified further by plating the 24 hour samples from all the wells onto IS agar plates to determine the bacterial concentration (CFU/mL). The percentage reduction was calculated as relative Log_10 _reduction from the starting bacterial inoculum concentration and plotted (Figure 4).

From the graph in Figure 4, stunted bacterial growth can be observed when maggot ES was used alone without ciprofloxacin (30% growth reduction). However, in combination with ciprofloxacin (at 80% and 60%) the maggot ES show significant reduction, of up to 50% from the starting inoculum concentration. When compared with ciprofloxacin at corresponding concentrations, the reduction observed was less than 5% or no reduction.

**Figure 5:**
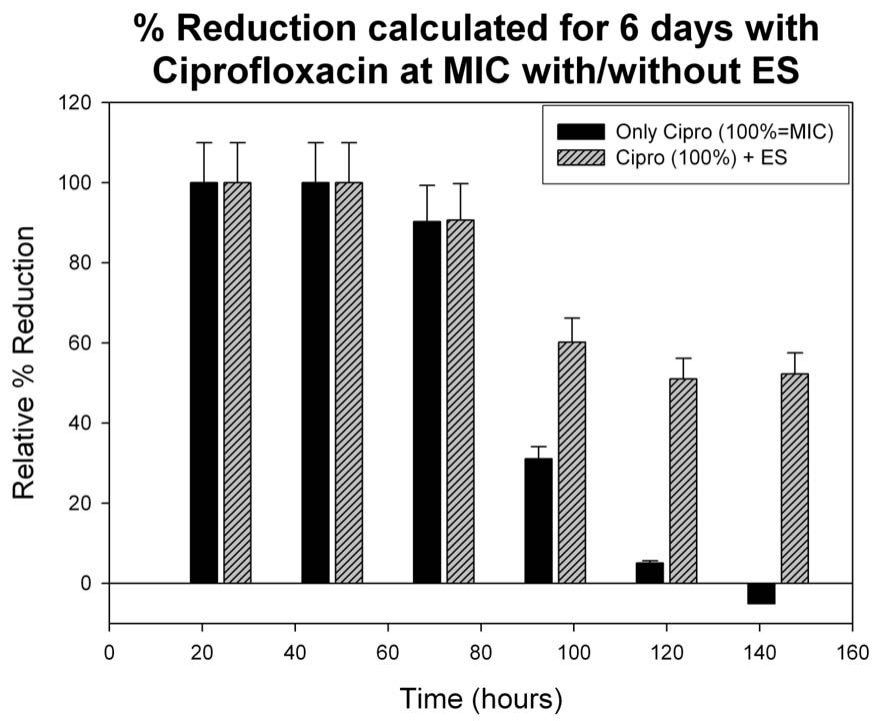
**Percentage reduction graph for 144 hr study**. Percentage reduction from the initial bacterial concentration at '0 hour' calculated every 24 hours of exposure of *S. aureus *to ciprofloxacin at MIC and maggot ES in combination with ciprofloxacin at MIC, for 144 hours (six days). The cell number was calculated over a range of dilutions and averaged. Each dilution was plated in triplicates for zero and 24 hours. The values of cell number obtained from each of the six wells of the micro-device were averaged for calculations.

The percentage reduction of bacterial number, calculated over the six day period of continuous exposure of *S. aureus *to ciprofloxacin alone and in combination with maggot ES, is summarized in Figure 5. It was observed form the initial trials, that addition of maggot ES was able to inhibit bacterial growth even after 72 hours of exposure, where ciprofloxacin alone failed to inhibit at MIC concentration. This can be clearly seen at the 144^th ^hour, where ciprofloxacin show no reduction in bacterial growth, while stunted growth with 52.26% (±10%) reduction was observed with ES addition. Similar reduction was seen on the subsequent days, in the strains exposed to maggot ES and ciprofloxacin combination.

## Discussion

In the previous study with *L. cuprina *maggot ES, the antibacterial activity that was observed, only showed stunted growth of *S. aureus *after 24 hours [20]. In this study, the qualitative and quantitative analysis of the ES revealed the presence of phelyacetaldehyde, a known antibacterial compound suggested previously to be secreted by the maggot midgut commensals [8]. The combination of ES with ciprofloxacin, as seen from the results, was able to enhance ciprofloxacin activity at sub-MIC levels. To some extent, the ES addition was also able to demonstrate a good prospect in delaying the process of development of resistant bacteria in the presence of antibiotic, for which adaptive resistance was observed otherwise. However, with the density of maggots used in the present study (50 maggots/100 μL of PBS), total bactericidal effects could not be detected as shown in previous studies with *L. sericata *maggots [11,15,16]. The methods adopted for collection of the ES in those studies were also different from the method described here with *L. cuprina *maggots.

Ciprofloxacin acts on *S. aureus *by disrupting the DNA replication mechanism. Its target, DNA gyrase is shown previously to undergo adaptive mutation when exposed to ciprofloxacin at near MIC concentrations. This may take place in as few as 4 days of drug exposure [22]. A point mutation in the subunits of the DNA topoisomerase enzyme (*grl*B gene), resulting in a resistant specie was reported by Stutandar *et. al.*[22], when they exposed *S. aureus *at near-MIC of ciprofloxacin. Similar point mutations have been demonstrated by others [27,28]. This kind of emergence of resistant bacterial species, due to antibiotic use, over use or abuse over time has become a major concern in the healthcare sector [29]. The emergence of multi-drug resistant MRSA in hospitals and chronic wounds, motivate the search for complementary, alternative and more effective antimicrobial solutions. Even stronger and newer antibiotics such as vancomycin, have given rise to resistant species over past few years [30]; coupled with the side effects and dose related toxicities associated with their use, it has encouraged research into alternative remedies.

Maggot secretions and excretions possess antibacterial activity against a wide range of pathogens as shown in previous studies and in their wound healing capabilities in biosurgery [2,11,15,16]. Bexfield and co-workers [11], purified and separated the maggot excretions based on their molecular weights by ultrafiltration. The low molecular weight fractions (5-10 kDa and <500 Da) of the ES demonstrated significant antibacterial activity against *S. aureus*. The fractions were also found to be heat stable, suggesting non proteineceous and non enzymatic factors. Recently, Cerovsky *et. al.*[19], isolated and purified a defensin peptide from the maggots of *L. sericata*, that coincided with the results of Bexfield *et. al. *This peptide, lucifensin, was reported to have molecular weight in the range of 5-10 kDa and exhibited antibacterial activity against a number of bacteria [19]. These low molecular weight defensin peptides are known to give insects their intrinsic ability to fight against pathogenic invaders as they possess antimicrobial properties [31]. They are also able to avoid emergence of resistance in some instances [32,33]. For this reason, maggot debridement therapy is particularly useful for the treatment of wounds infected with hospital-acquired MRSA. The results have been demonstrated by Beasley and Hirst in their study of wounds infected by multi-drug resistant MRSA [8].

Several mechanisms of wound disinfection by maggots have been proposed in the past. One of the suggested mechanisms is by simple mechanical irrigation of wound by increased exudate, caused by ingestion of liquefied necrotic tissues by the larvae. This results in wound lavage and dilution of bacterial concentration over the wound [1,8]. Some have suggested excretion of ammonia that increases the pH of the wound, making it unfavourable for many bacterial species to survive. The ES collected in this study also exhibited alkaline pH value, supporting the previous findings. Some have even suggested ingestion of bacteria by the larvae during wound irrigation, followed by their digestion as they pass through the digestive tract of the maggots. Mumcuoglu *et. al.*[17], in their work, demonstrated the destruction of ingested bacteria in the midgut of maggots. Maggots are also known to secrete antibacterial compounds such as phelyacetaldehyde and phenylacetic acid, that are secreted by the midgut commensals of maggots, *P. Mirabilis *[8,34].

Besides identification of the antibacterial factors in the ES, the current study was also able to show potentiation of ciprofloxacin activity at sub-MIC concentrations in combination with maggot ES in the 24 hour study. In general, the use of antibiotics in combination from the start may help to reduce the events of development of resistance in a pathogen [35]. Moreover, hypothetically, if the density of the ES preparation could be increase there would be improved bactericidal effects. However, practical constraints to rear large number of maggots limited that possibility. Therefore, a combination therapy could be potentially useful and considered in appropriate situations. The further study on survivability of *S. aureus *in presence of both ciprofloxacin and insect extract for 144 hours, demonstrated high growth reduction compared to ciprofloxacin exposure alone. The adaptive resistance could be observed in the fourth day of exposure to ciprofloxacin alone (after 96 hours), where less reduction in bacteria number (~ 30% reduction) was seen. In the following days, the reduction in the ciprofloxacin exposed strain reduced to 5%, followed by no reduction. Whereas, with added ES, the reduction was maintained at more than 50% throughout the six days of the study. This clearly suggested an inhibition of adaptive resistance development in the exposed bacteria by ES addition as a potentiating or resistance modifying agent. Further determination of resistance profiles of MSSA by molecular and other methods, under the influence of the combination would give detailed insights into the potentiating activity of the insect extract.

## Conclusions

The present study shows that ES extracted from maggots of *L. cuprina *in combination with ciprofloxacin antibiotic at concentrations of 80 and 60% of MIC, demonstrate up to 50% reduction in bacterial number from the starting inoculum concentration, after 24 hours of exposure. The outcome of the 144 hours of exposure of the bacteria to the ES-ciprofloxacin combination, suggested a potential delay in the development of adaptive resistance to the antibiotic. The results clearly suggest a potentiation of the antibiotic activity. The ES was analyzed by GC-MS, and revealed the presence of antibacterial compound, phenylacetaldehyde and had an alkaline pH. Both these findings support the antibacterial activity of the ES. The study was carried out in a system designed to perform separation and mixing of two fluids in various concentrations whilst performing culturing, sustaining and monitoring cells in a favorable growth environment.

## List of abbreviations

ATCC: American type culture collection; CFU: colony forming unit; ES: Excretion/secretion; GC: Gas chromatograph; IS: Iso-sensitist; *L. cuprina: Lucilia cuprina; L. sericata: Lucilia sericata; *MIC: minimum inhibitory concentration; MRSA: Methecillin resistant *S. aureus; *MS: Mass spectrometer; MSSA: Methecillin sensitive *S. aureus; *OD: Optical density; PBS: Phosphate buffer saline; *P. mirabilis: Proteus mirabilis; S. aureus: Staphylococcus aureus*

## Competing interests

The authors declare that they have no competing interests.

## Authors' contributions

SA carried out the experimental study on *S. aureus *and was responsible for data collection and analysis. LCS and SA participated in drafting the manuscript. LCS also critically revised the manuscript draft for important intellectual content and approved the current version to be published. CB and his team helped in rearing and extraction from maggots and related activities. All authors read and approved the final manuscript.
